# The effects of human parvovirus VP1 unique region in a mouse model of allergic asthma

**DOI:** 10.1371/journal.pone.0216799

**Published:** 2019-05-14

**Authors:** Shyh-Ren Chiang, Chia-Yun Lin, Der-Yuan Chen, Hui-Fang Tsai, Xin-Ci Lin, Tsai-Ching Hsu, Bor-Show Tzang

**Affiliations:** 1 Department of Internal Medicine, Chi Mei Medical Center, Tainan, Taiwan, R.O.C; 2 Department of General Science, Chia Nan University of Pharmacy and Science, Tainan, Taiwan, R.O.C; 3 Institute of Biochemistry, Microbiology and Immunology, Chung Shan Medical University, Taichung, Taiwan, R.O.C; 4 Rheumatology and Immunology Center, China Medical University Hospital, Taichung, Taiwan, R.O.C; 5 Rheumatic Diseases Research Center, Department of Medical Research, China Medical University Hospital, Taichung, Taiwan, R.O.C; 6 School of Medicine, China Medical University, Taichung, Taiwan, R.O.C; 7 Department of Medical Laboratory and Biotechnology, Chung Shan Medical University, Taiwan, R.O.C; 8 Clinical Laboratory, Chung Shan Medical University Hospital, Taichung, Taiwan, R.O.C; 9 Immunology Research Center, Chung Shan Medical University, Taichung, Taiwan, R.O.C; 10 Department of Biochemistry, School of Medicine, Chung Shan Medical University, Taichung, Taiwan, R.O.C; Forschungszentrum Borstel Leibniz-Zentrum fur Medizin und Biowissenschaften, GERMANY

## Abstract

Evidence has indicated that viral infection increases the risk of developing asthma. Although the association of human parvovirus B19 (B19V) or human bocavirus (HBoV) with respiratory diseases has been reported, little is known about the influence of the B19V-VP1u and HBoV-VP1u proteins on the symptoms of asthma. Herein, we investigated the systemic influence of subcutaneously injected B19V-VP1u and HBoV-VP1u recombinant proteins in an OVA-sensitized asthmatic mouse model. A significantly higher Penh ratio and IgE level were detected in the serum, bronchoalveolar lavage fluid (BALF) and the supernatant of a lymphocyte culture from mice treated with HBoV-VP1u or B19V-VP1u than in a lymphocyte culture from OVA-sensitized mice. Significantly higher levels of serum and BALF IgE, total IgG, IgG1, OVA-specific IgE and OVA-specific IgG1 were detected in mice treated with HBoV-VP1u or B19V-VP1u than in OVA-sensitized mice. Conversely, a significantly lower IgG2a level was detected in mice from the HBoV-VP1u or B19V-VP1u groups than in mice from the OVA group. The mice treated with HBoV-VP1u or B19V-VP1u exhibited more significant lung inflammatory indices, including elevated serum and BALF IL-4, IL-5, IL-10 and IL-13 levels; BALF lymphocyte, neutrophil and eosinophil counts, MMP-9 and MMP-2 activity; and the amount of lymphocyte infiltration, relative to those in the control mice or in those sensitized with OVA. These findings demonstrate that the subcutaneous injection of HBoV-VP1u or B19V-VP1u proteins in OVA-sensitized mice result in elevated asthmatic indices and suggest that human parvoviruses may increase the risk of developing airway inflammation in a mouse model of asthma.

## Introduction

Asthma is a chronic lung disease that inflames and narrows the airways of the lungs. The major symptoms of asthma include coughing, shortness of breath, and chest tightness [1–3]. The causes of asthma are complex and involve interactions among multiple genetic and environmental factors. Extensive evidence has revealed that viral infection early in life could be a primary environmental risk factor for the development of asthma [4–5]. Many studies have also indicated that viral infection could be a major trigger of wheezing in infants and of the exacerbation of asthma in older children [4–5]. Notably, viral infections are detected in up to 85% of young patients with wheezing or asthma [6].

Both human parvovirus B19 (B19V) and human bocavirus (HBoV) belong to *Parvoviridae*, members of which contain the VP1 unique (VP1u) region. The VP1u region of B19V, called B19V-VP1u, contains 227 amino acids, and the VP1u region of HoBV, called HBoV-VP1u, contains 129 amino acids. Notably, the VP1u regions of both B19V and HBoV have the motif of and exhibit activity of secreted phospholipidase (sPLA2), which is strongly associated with the ability to infect and induce inflammation in host cells [7–9]. Notably, human B19V and HBoV have been reported as respiratory viruses and are closely related to the risk of various respiratory diseases [10–14]. Many studies have also indicated that both B19V and HBoV are associated with asthma in children [10–18].

Since the VP1u of human parvoviruses are known to play crucial roles in the viral infectivity and in the induction of inflammatory responses in infected hosts [7–9], the current study investigated the effects of B19V-VP1u and HBoV-VP1u on the development of asthma. Herein, we used a nonlocal viral infection method by subcutaneously injecting B19V-VP1u or HBoV-VP1u recombinant proteins in OVA-sensitized mice to mimic the systemic effect of parvovirus infection to study the effect of these viruses on asthmatic symptoms.

## Materials and methods

### Preparation of recombinant human HBoV-VP1u and B19-VP1u proteins

The recombinant B19V-VP1u and HBoV-VP1u proteins were prepared as described previously [7]. Briefly, the DNA fragments encompassing B19V-VP1u and HBoV-VP1u were obtained by the polymerase chain reaction (PCR), respectively [19–20]. Next, the B19V-VP1u and HBoV-VP1u DNA fragments were separately ligated into pET-32a vector (Novagene, Cambridge, MA). The ligatants, as called pET32a-B19V-VP1u and pET32a-HBoV-VP1u, were then transformed into Escherichia coli BL21-DE3 competent cells (Invitrogen, Carlsbad, CA). The expressions of B19V-VP1u and HBoV-VP1u recombinant proteins were induced by addition of IPTG (1 mM) and purified with Ni-NTA spin columns (Qiagen, Chatsworth, CA, USA) and PureProteome Nickel Magnetic Beads (EMD Millipore, CA, USA). The purified B19V-VP1u and HBoV-VP1u recombinant proteins were analyzed with HPLC and the purities of the purified recombinant proteins were 98.6% and 98.1%, respectively. Endotoxin tests were conducted using the Limulus Amebocyte Lysate Endochrome Assay (Charles River Laboratories, Inc., Charleston, SC, USA). The endotoxin levels were found to be below the detection limit [0.25 endotoxin unit (EU)/ml] for all recombinant protein preparations at the concentrations used in the present study.

### Immunoblotting

Immunoblotting was performed as described elsewhere [19]. Briefly, purified recombinant VP1u proteins of HBoV and B19V were separated in a 12% SDS-PAGE and electrophoretically transferred to nitrocellulose membrane (Bio-Rad, Hercules, CA, USA). After blocking with 5% non-fat dry milk, antibodies against Histidine tag (6xHis) mouse monoclonal antibody (Invitrogen, CA, USA) were incubated with the membrane for 1.5 hours with gentle agitation. The membrane was then washed and incubated with alkaline phosphatase conjugated goat anti-mouse IgG antibodies (Sigma, Saint Louis Mo, USA) at a dilution of 1/5000. The substrate nitroblue tetrazolium/ 5-bromo-4-chloro-3 indolyl phosphate (NBT/BCIP) (USB Corporation, Ohio, USA) was used to detect antigen-antibody complexes.

### sPLA2 catalytic activity

The sPLA2 activity was detected as described elsewhere [19]. A colorimetric assay (sPLA2 Activity Kit; Cayman Chemical) was adapted to measure sPLA2 activities of recombinant B19V-VP1u and HBoV-VP1u proteins according to the manufacturer’s instructions, with dynamic colorimetric measurements (the optical density at 414 nm) determined every minute for 10 min. Results are revealed as micromoles per minute per milliliter.

### Ethics and animal

Twenty female BALB/c mice at six-week old were purchased from the National Laboratory Animal Center, Taiwan. Approval for this study was obtained from the Institutional Animal Care and Use Committee of Chung Shan Medical University, Taichung, Taiwan (No. 1616). All animals were housed in a room with a temperature-, humidity- and light-controlled environment and had free access to water and standard laboratory chow (Lab Diet 5001; PMI Nutrition International Inc., Brentwood, MO, USA). At the age of eight-week, the mice were randomly divided into four groups, including PBS (control), OVA, HBoV-VP1u and B19V-VP1u groups, for further investigation.

### The mouse model of allergic asthma

The mice from OVA, HBoV-VP1u and B19V-VP1u groups were immunized by intraperitoneal (i.p.) injections of 0.2mg OVA (grade V; Sigma Chem. Co., St Louis, USA) emulsified in 4mg of aluminium hydroxide (Alum) (Imject Alum Adjuvant, Pierce Chemical, Rockford, IL, USA) in a total volume of 200 μl PBS on Days 0, 14 and 29. The mice from PBS group were received an i.p. injection of 0.2 mL PBS complexed with alum as unsensitized controls. On day 34 and 41, the mice from HBoV-VP1u and B19V-VP1u groups were subcutaneously (s.c.) injected with 20 μg of HBoV-VP1u and B19V-VP1u recombinant proteins, respectively. The mice from PBS and OVA groups were subcutaneously (s.c.) injected with PBS on day 34 and 41. The Intranasal (i.n.) OVA challenge was performed in mice from OVA, HBoV-VP1u and B19V-VP1u groups by giving a dose of 40 μg OVA in 25 μL PBS on day 44 to 48. In the control group, unsensitized mice received 25 μL of PBS i.n. on day 44 to 48.

### Airway responsiveness (AHR)

Airway responsiveness (AHR) of mice was measured through whole-body barometric plethysmography (Buxco Electronics, New York, USA) to record the enhanced pause (Penh). Mice were exposed to the inhalation of PBS and subsequent increasing doses of methacholine (Sigma, St Louis, MO, USA, 10, 20, and 30 mg/ml). Each nebulization lasted for 2 min and records were taken for 2 min after nebulization. Every aerosol was separated by a 15-min recovery period in order to allow airway Penh to return to the baseline level. Penh results were averaged and expressed as fold difference over baseline.

### Bronchoalveolar lavage fluid (BALF) and cell count

The bronchoalveolar lavage (BAL) of mice was performed after exsanguinations and the BAL fluid (BALF) and BAL cells were isolated as described elsewhere [21]. Briefly, BAL of the left lung was performed by ligating the right lung to the right main-stem bronchus. The BALF was centrifuged at 1500 rpm for 5 minutes at 4°C. The BALF cells were stained with Liu’s stain and a hemocytometer was used to determine the total leukocyte counts. Moreover, the BALF cells was sprayed on slides with a cytospin device (Cytospin Cytocentrifuge, Thermo shandon) and the slides were stained with Liu’s stain (Sigma, St. Louis, MO, United States). Differential counts were performed in a blinded fashion by counting 200 cells under a light microscope.

### Spleen cells isolation

Mice were sacrificed by CO_2_ asphyxiation. The spleens were harvested and placed in cold Roswell Park Memorial Institute (RPMI)-1640 medium supplemented with 10% fetal bovine serum (Invitrogen, Carlsbad, CA, USA), 10 mg/ml gentamycin, 2 mM L-glutamine, and 0.1 mM 2-mercaptoethanol. Red blood cells were lysed in red blood cell lysing buffer (Bio-Rad, Hercules, CA, USA) on ice according to manufacturer's instruction. The splenocytes were then incubated with RPMI-1640 medium supplemented with 10% fetal bovine serum in at 37°C and 5% CO^2^ in an incubator.

### The serum and BALF antibodies

The serum and BALF antibodies, including IgE, IgG1 and IgG2a, were determined by using specific ELISA kits according to the manufacturer’s instruction (eBioscience, San Diego, CA, USA). Sera samples were assayed at a dilution of 1:200 for IgE analysis, 1:10000 for IgG1 analysis, and 1:100 for IgG2a analysis, and BALF samples were assayed at a dilution of 1:2 for IgE analysis, 1:50 for IgG1 analysis, and 1:1 for IgG2a analysis. Finally, the absorbance (OD) of the wells at 450 nm was read.

### Detection of BALF OVA-specific IgE, IgG1 and IgG2a

The levels of OVA-specific IgE, IgG1 and IgG2a in BALF were measured with a modified ELISA assay (BETHYL Laboratories Inc., TX, USA) as described elsewhere [22]. Briefly, ELISA plates were coated with 200μg/ml of OVA in 0.1M NaHCO_3_ (pH 9.6) and incubated at 4°C for overnight. After washing with 1x PBS, the plates were then blocked with 1% bovine serum albumin (BSA) in 1 × PBS (blocking buffer) for another three hours at room temperature. Then, BALF were serially diluted in blocking buffer and incubated at 4°C for overnight. Sequentially, the plates were incubated with horseradish peroxidase-conjugated anti-mice IgE, IgG1 or IgG2a antibodies at 4°C for overnight. To detect the binding reactivity, tetramethylbenzidine were added and the absorbance at 450nm was measured.

### Gel zymography

The activities of matrix metalloproteinase (MMP)-9 and MMP-2 were detected by gelatin zymography. Briefly, ten microliters of BALF from each experiment groups were separated by an 8% sodium dodecyl sulfate-polyacrylamide gel electrophoresis (SDS-PAGE) gel polymerized with 0.1% gelatin. After washing with 2.5% Triton X-100 buffer, the gel was then soaked in the reaction buffer (40 mM Tris-HCl, 10 mM CaCl_2_, and 0.02% (w/v) NaN3) for 16h. Gelatinolytic activity was visualized by staining the gels with 0.5% Coomassie brillant blue R-250 and quantified by densitometry (Alpha-Imager 2200; ProteinSimple, San Jose, CA, USA).

### Haematoxylin-eosin staining

The lung tissues of animals from each group were excised and soaked in formalin and covered with wax. Slides were prepared by deparaffinization and dehydration [14]. After passing through a series of graded alcohols (100%, 95% and 75%), the slides were then dyed with haematoxylin. The slides were then rinsed with water and soaked with 85% alcohol, 100% alcohol I and II for 15 mins. At the end, they were soaked with Xylene I and Xylene II. Photomicrography was performed with Zeiss Axiophot microscopes. For quantification, the number of infiltrated lymphocytes was counted in 5 randomly selected fields of a section slide. All measurements were made using at least 3 section slides from 3 independent animals.

### Enzyme-linked immunosorbent assay (ELISA)

The serum and BALF interleukin (IL)-4, IL-5, IL-10, IL-13 and IFN-γ levels were measured with Bio-Plex mouse cytokine assay kits and Bio-Plex manager software version 3.0 using five parametric curve fitting (Bio-Rad, Hercules, CA, USA) [23].

### Statistical analysis

The GraphPad Prism 5 software (GraphPad Software, CA) by one-way analysis of variance (One-way ANOVA) followed by Tukey multiple-comparisons test was used to perform the statistical analyses. All data were shown as mean ± SEM and verified at least 3 independent experiments. A P value less than 0.05 was considered statistically significant.

## Results

### Preparation of recombinant HBoV-VP1 u and B19V-VP1u proteins

**Fig 1:**
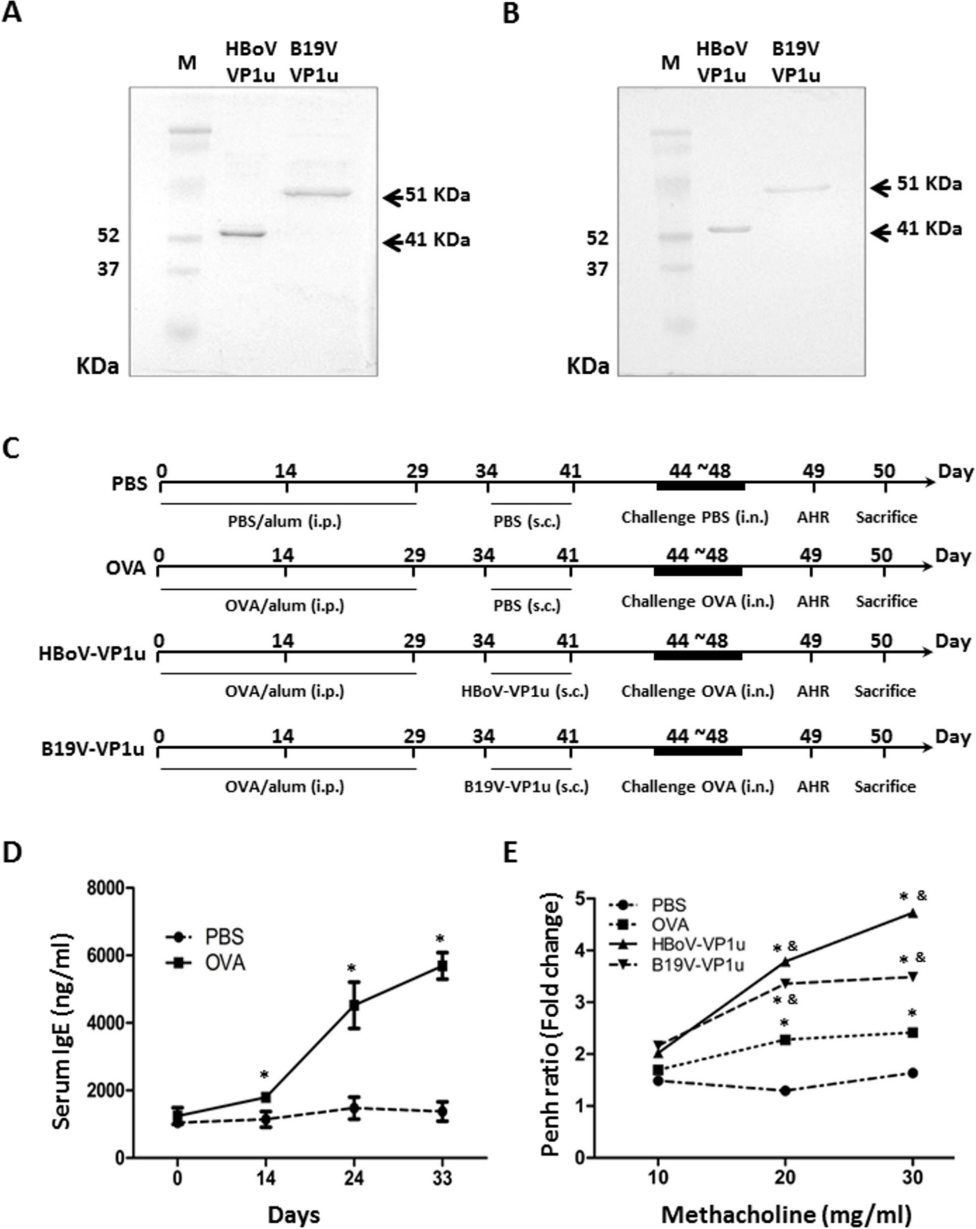
The mouse models of allergic asthma. (A) The purified HBoV-VP1u and B19V-VP1u recombinant was observed on SDS-PAGE and (B) detected with immunoblotting by probing with antibodies against histidine tag (6x His). (C) The protocols used for the four mouse models of allergic asthma. (D) Serum level of IgE in mice from PBS and OVA groups on day 0, 14, 24 and 33. (E) The fold change of enhanced pause (Penh) of mice. Values are mean ± SD. Similar results were observed in three repeated experiments. * and & indicate significant difference, p<0.05, relative to PBS and OVA groups, respectively. (n = 5 for each group).

Recombinant HBoV-VP1u and B19V-VP1u proteins were prepared as described in the Materials and Methods section. Fig 1A shows the SDS-PAGE results for the purified recombinant HBoV-VP1u and B19V-VP1u proteins, and Fig 1B shows the immunoblotting results that were obtained by probing with antibodies against the His-tag. HBoV-VP1u and B19V-VP1u recombinant proteins were also analyzed to measure their PLA2 activity. As a positive control, bvPLA2 exhibited a sPLA2 enzymatic activity of 0.243±0.037 μmol/min/mL. Significant sPLA2 activities were detected in both HBoV-VP1u and B19V-VP1u recombinant proteins, with sPLA2 activities of 0.046±0.006 μmol/min/mL and 0.013±0.001 μmol/min/mL, respectively. No sPLA2 activity was detected in the B19V-VP2 or B19V-NS1 recombinant protein (Table 1).

**Table 1:** Secreted phospholipase A2 (sPLA2) activity of recombinant B19V-VP1u and HBoV-VP1u proteins.

Proteins	sPLA2 activity (μmol/min/mL)
bvPLA2 (10 ng)	0.243±0.037
B19V-VP1u (400 ng)	0.013±0.001
HBoV-VP1u (400 ng)	0.046±0.006
B19V-VP2 (400 ng)	ND
B19V-NS1 (400 ng)	ND

bvPLA2: sPLA2 from bee venom PLA2 control; B19V: human parvovirus B19; HBoV: human bocavirus; VP1u: VP1 unique region; ND: non-detected

### Induction of allergic asthma

Fig 1C shows the allergic asthma model. Mice were intraperitoneally injected with PBS with alum or were sensitized to OVA with alum (200 μg OVA in 0.2 mL PBS) on days 0, 14 and 29 before undergoing an intranasal (i.n.) OVA challenge on days 44 to 48. On days 34 and 41, the mice from the PBS and OVA groups were subcutaneously (s.c.) injected with PBS, and those from the HBoV-VP1u and B19V-VP1u groups were subcutaneously (s.c.) injected with HBoV-VP1u and B19V-VP1u recombinant proteins, respectively. The airway responsiveness (AHR) was measured on day 49, and all animals were sacrificed on day 50. To confirm the induction of IgE, cheek blood was obtained from mice in the PBS and OVA groups on days 0, 14, 24 and 33. Significantly higher serum IgE levels were detected in the serum of mice from the OVA groups on days 14, 24 and 33 (Fig 1D) compared to those in the PBS groups. The lung function of the mice was quantified by measuring the airway response in enhanced pause (Penh) vs. the methacholine concentration. The Penh ratio was significantly higher in mice that were sensitized with OVA, HBoV-VP1u and B19V-VP1u than in those that were sensitized with PBS (Fig 1E) upon challenge with methacholine at a concentration of 20 mg/mL and higher. Moreover, a significantly higher Penh ratio was observed in mice that were sensitized with HBoV-VP1u and B19V-VP1u than in those that were sensitized with OVA upon challenge with methacholine at a concentration of at least 20 mg/mL.

### HBoV-VP1u and B19V-VP1u increase asthmatic-related antibodies in OVA-sensitized mice

**Fig 2:**
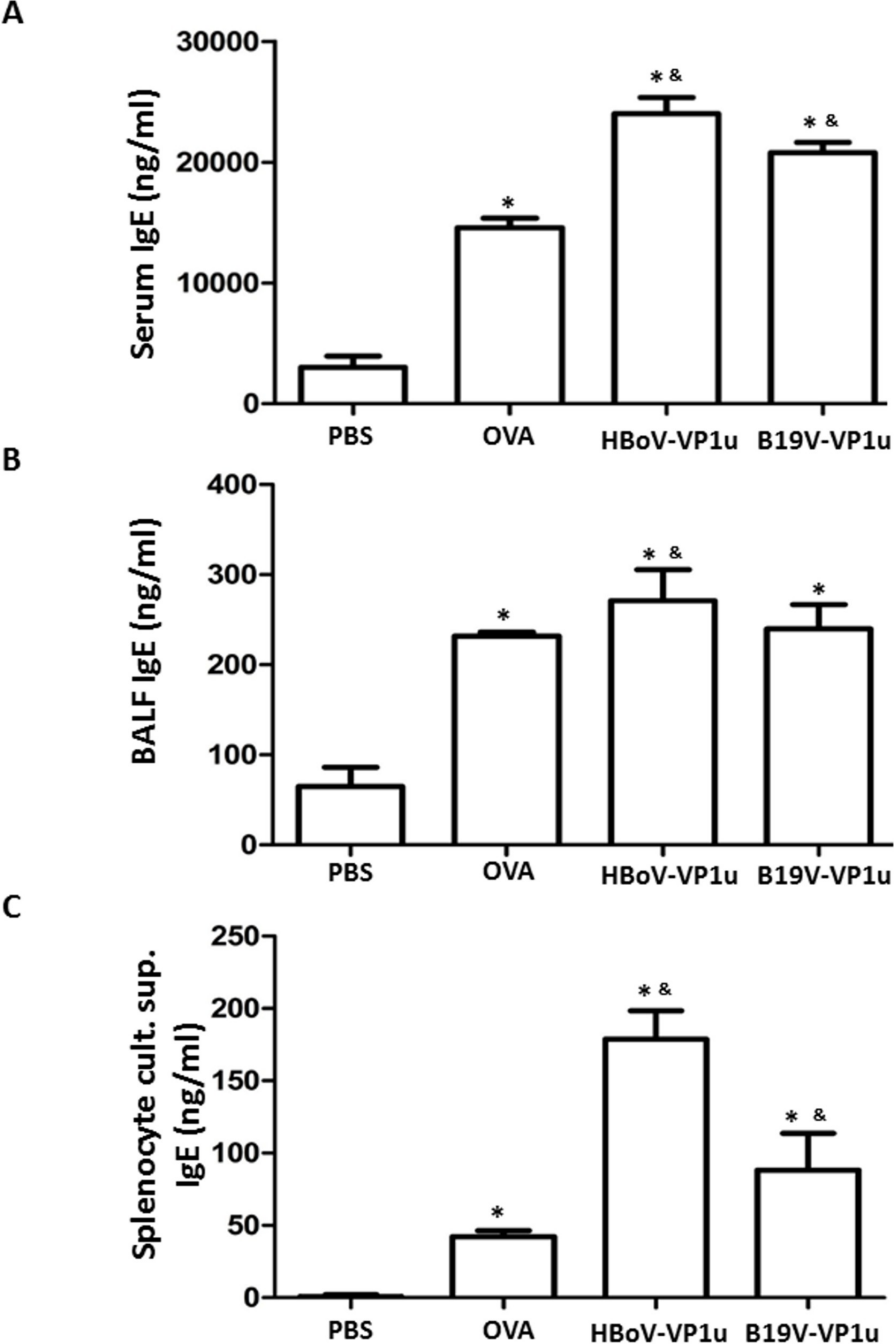
The level of IgE. The specific anti-OVA IgE was measured in (A) serum, (B) BALF and (C) supernatant of splenocytes culture in mice from PBS, OVA, HBoV-VP1u and B19V-VP1u groups. Values are mean ± SD. Similar results were observed in three repeated experiments. * and & indicate significant difference, p<0.05, relative to PBS and OVA groups, respectively. (n = 5 for each group).

**Fig 3:**
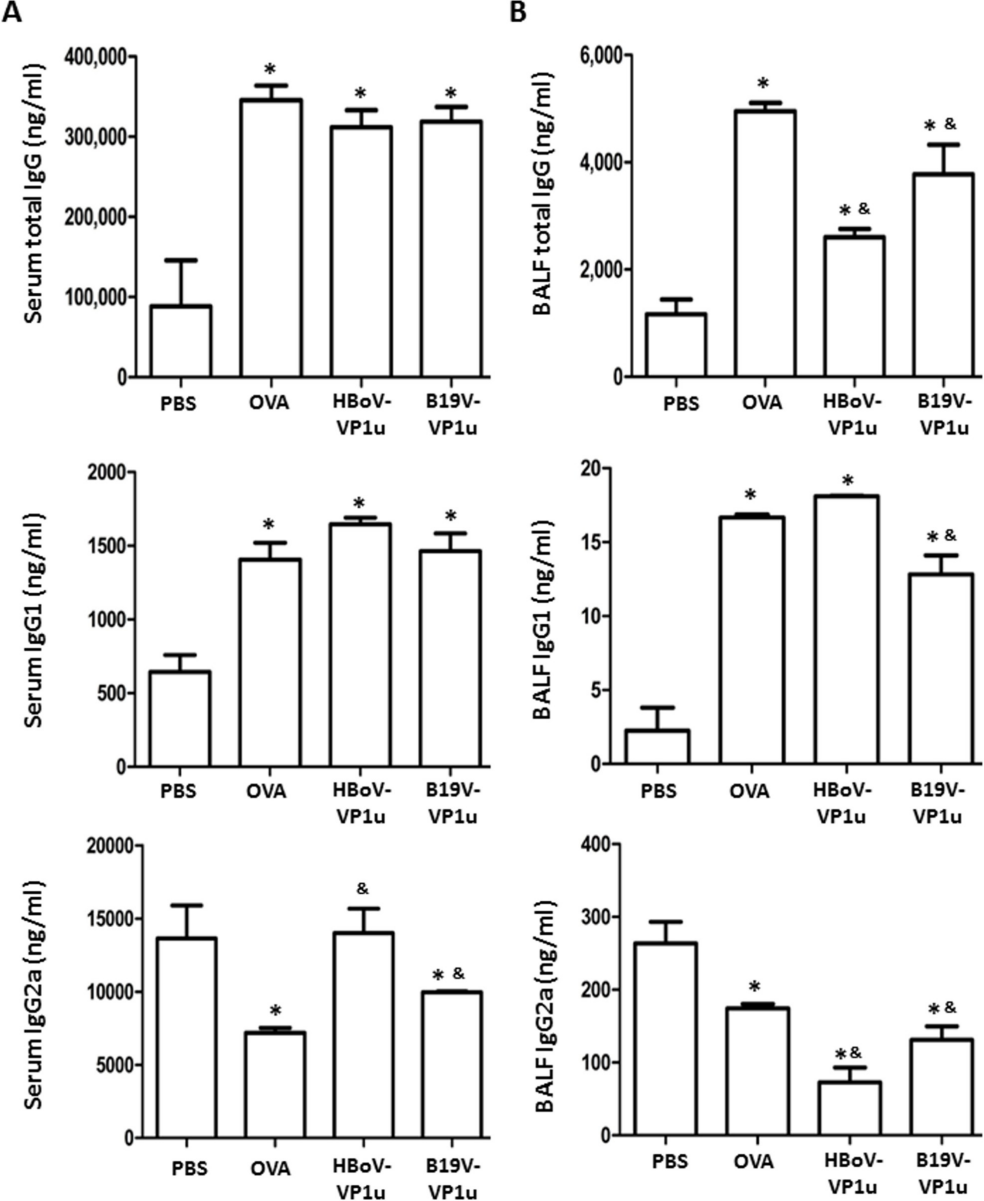
The levels of IgG subtypes. The total IgG, IgG1 and IgG2a in (A) serum and (B) BALF was measured in mice from PBS, OVA, HBoV-VP1u and B19V-VP1u groups. Values are mean ± SD. Similar results were observed in three repeated experiments. * and & indicate significant difference, p<0.05, relative to PBS and OVA groups, respectively. (n = 5 for each group).

**Fig 4:**
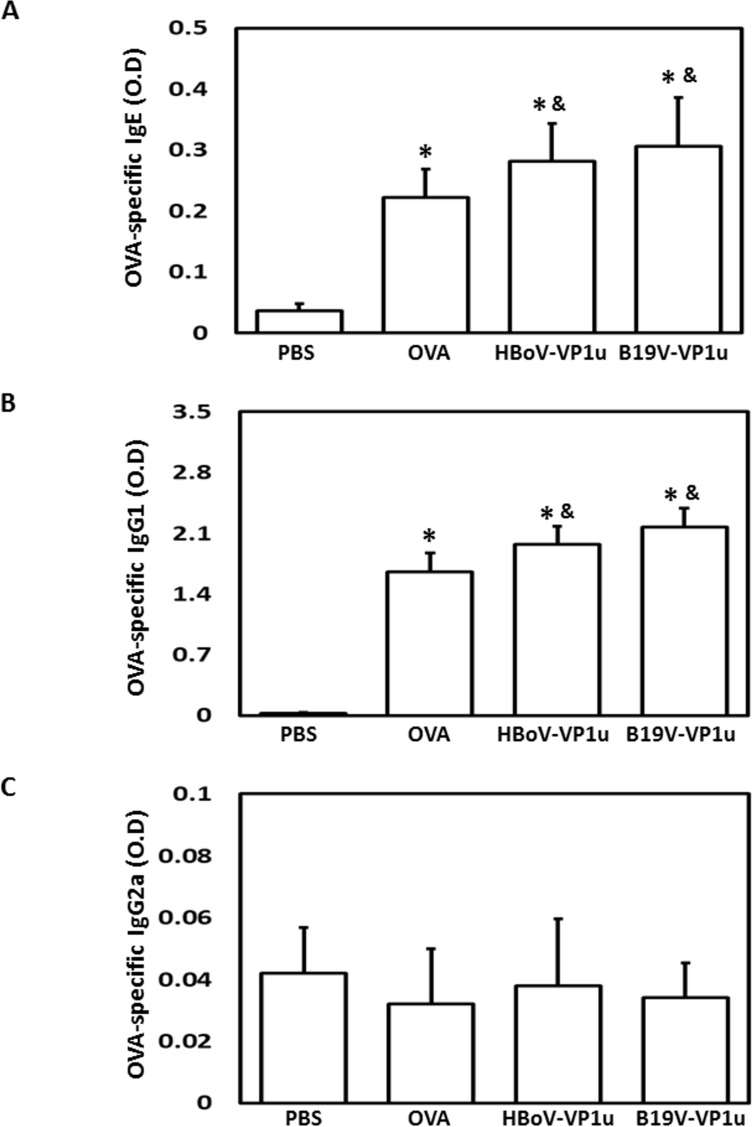
OVA-specific antibodies in BALF. The levels of OVA-specific (A) IgE, (B) IgG1 and (C) IgG2a in BALF were detected in mice from PBS, OVA, HBoV-VP1u and B19V-VP1u groups. Values are mean ± SD. Similar results were observed in three repeated experiments. * and & indicate significant difference, p<0.05, relative to PBS and OVA groups, respectively. (n = 5 for each group).

To detect the levels of IgE, total IgG, IgG1 and IgG2a in OVA-sensitized mice, an ELISA assay was conducted. Significantly higher levels of IgE were detected in the serum, BALF and supernatant of the splenetic lymphocyte culture of mice from the OVA, HBoV-VP1u and B19V-VP1u groups than in those from the PBS group (Fig 2). Notably, significantly higher levels of IgE were observed in the serum and supernatant of the splenetic lymphocyte culture of mice from the HBoV-VP1u and B19V-VP1u groups than in those of mice from the OVA group (Fig 2). Significantly higher levels of total IgG and IgG1 were detected in both the serum and the BALF of mice from the OVA, HBoV-VP1u and B19V-VP1u groups than in those of the mice from the PBS group (Fig 3). A significantly lower level of serum IgG2a was detected only in mice from the OVA and B19V-VP1u groups compared to that in mice from the PBS group (Fig 3A). Additionally, a significantly lower BALF IgG2a level was observed in mice from the OVA, HBoV-VP1u and B19V-VP1u groups than in that of the mice from the PBS group (Fig 3B). Significantly higher serum IgG2a levels were observed in mice from the HBoV-VP1u group than in mice from the OVA group (Fig 3A). Significantly lower BALF total IgG and IgG2a levels were observed in mice from the HBoV-VP1u and B19V-VP1u groups than in mice from the OVA group (Fig 3B). A significantly lower BALF IgG1 level was observed in mice from the B19V-VP1u group than in mice from the OVA group (Fig 3B). The levels of OVA-specific IgE, IgG1 and IgG2a in the BALF were also measured (Fig 4). Significantly higher BALF OVA-specific IgE and IgG1 levels were detected in mice from the OVA, HBoV-VP1u and B19V-VP1u groups than in the PBS group (Fig 4A and 4B). Notably, significantly higher BALF OVA-specific IgE and IgG1 levels were observed in mice from the HBoV-VP1u and B19V-VP1u groups than in the OVA group (Fig 4A and 4B). No significant difference in BALF OVA-specific IgG2a was found among the groups (Fig 4C).

### HBoV-VP1u and B19V-VP1u increase lung inflammation in OVA-sensitized mice

**Fig 5:**
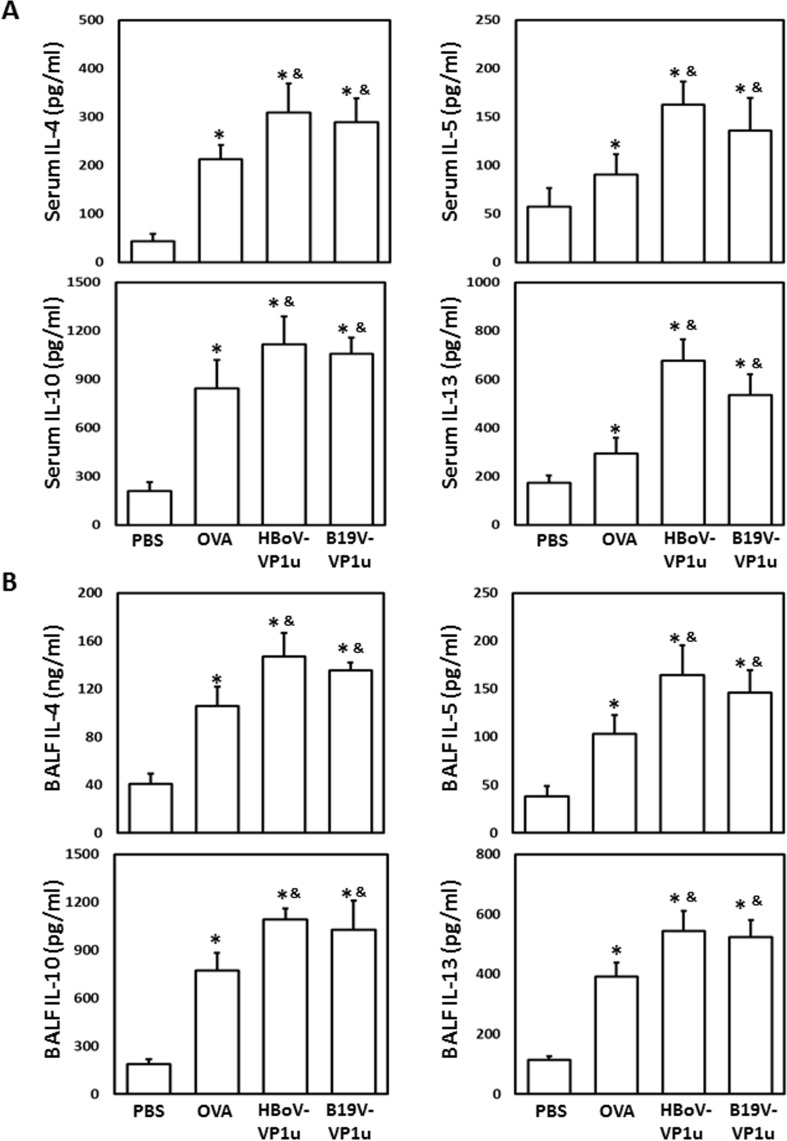
The levels of cytokines. The levels of IL-4, IL-5, IL-10 and IL-13 in (A) serum and (B) BALF of mice from PBS, OVA, HBoV-VP1u and B19V-VP1u groups were measured with ELISA kits. Values are mean ± SD. Similar results were observed in three repeated experiments. * and & indicate significant difference, p<0.05, relative to PBS and OVA groups, respectively. (n = 5 for each group).

**Fig 6:**
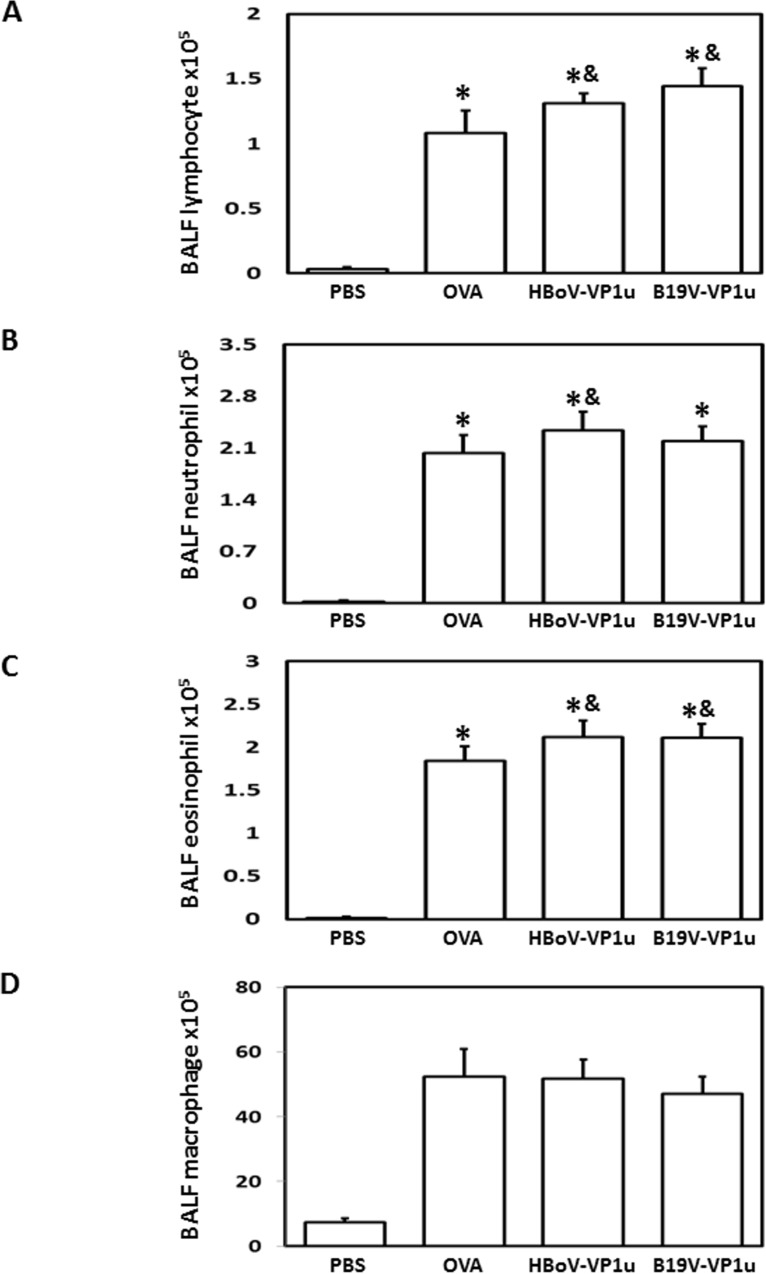
Differential cell count in BALF. The BALF of mice from PBS, OVA, HBoV-VP1u and B19V-VP1u groups were harvested and percentages of (A) lymphocyte, (B) neutrophil, (C) eosinophil and (D) macrophage were calculated. Values are mean ± SD. Similar results were observed in three repeated experiments. * and & indicate significant difference, p<0.05, relative to PBS and OVA groups, respectively. (n = 5 for each group).

**Fig 7:**
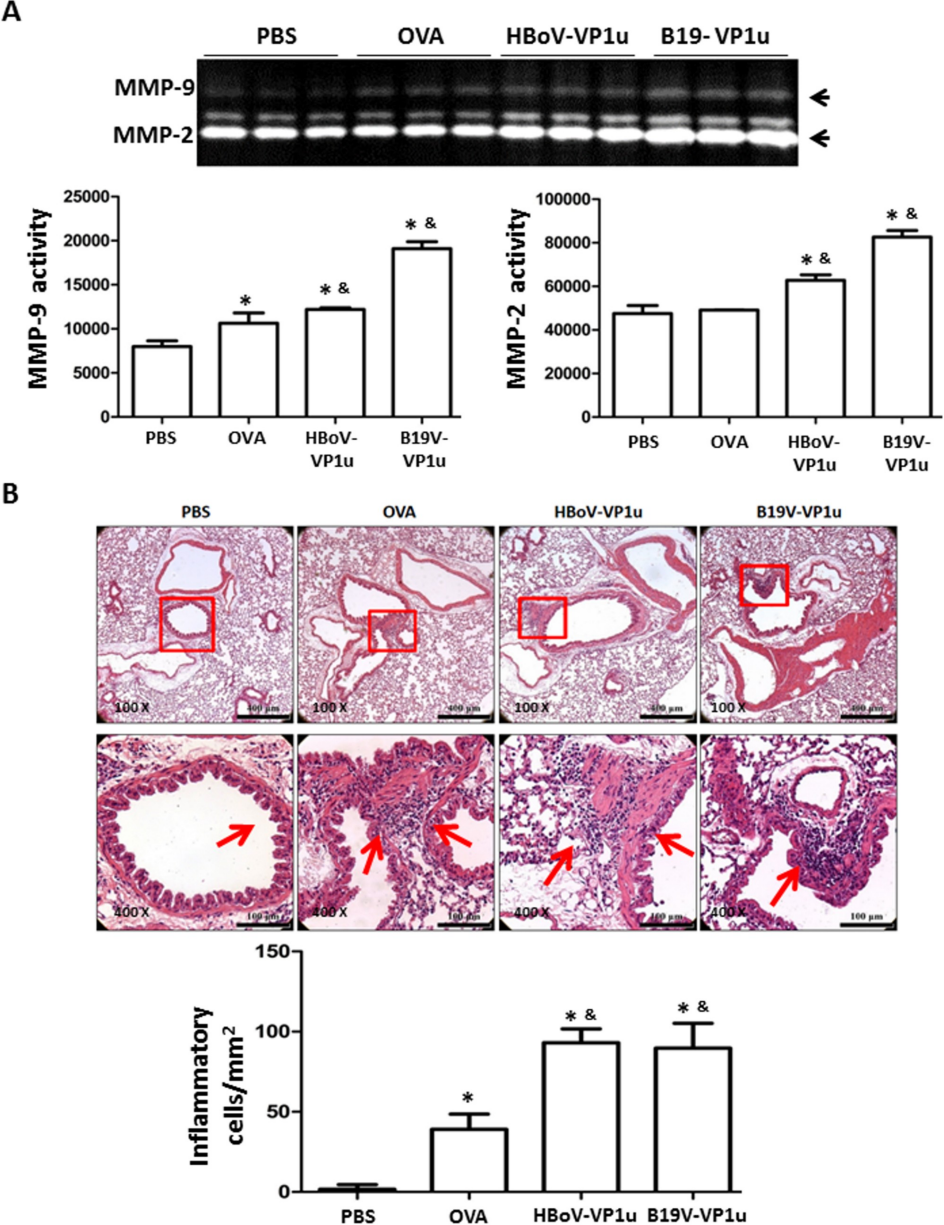
Allergen-induced airway inflammation. Lung tissues were obtained on Day 50 from the mice of PBS, OVA, HBoV-VP1u and B19V-VP1u groups. (A) MMP-9 and MMP-2 activity were detected by gelatin zymography. Quantified results of MMP-9 and MMP-2 activities were shown in the lower panel. (B) The sections of lung tissues were stained with hematoxylin and eosin and revealed with a magnification ratio of 1:100 (upper panel) or 1:400 (lower panel). Amplified images were shown in the lower panel of each section. The lymphocyte infiltration was indicated by an arrow. Values are mean ± SD. Similar results were observed in three repeated experiments. * and & indicate significant difference, p<0.05, relative to PBS and OVA groups, respectively. (n = 5 for each group).

To evaluate the effect of HBoV-VP1u and B19V-VP1u on lung inflammation in OVA-sensitized mice, serum and BALF cytokine levels were measured. Significantly higher levels of IL-4, IL-5, IL-10 and IL-13 were detected in mice from the OVA, HBoV-VP1u and B19V-VP1u groups than in mice from the PBS group (Fig 5). Significantly higher levels of IL-4, IL-5, IL-10 and IL-13 were observed in mice from the HBoV-VP1u and B19V-VP1u groups than in mice from the OVA group (Fig 5). Differential cell counts in the BALF were also performed. Significantly higher numbers of lymphocytes, neutrophils, eosinophils and macrophages were observed in the BALF of mice from the OVA, HBoV-VP1u and B19V-VP1u groups than in the PBS group (Fig 6). Significantly higher numbers of BALF lymphocytes and eosinophils were observed in mice from the HBoV-VP1u and B19V-VP1u groups than in the OVA group (Fig 6A and 6C). A significantly higher number of BALF neutrophils was observed in mice from the HBoV-VP1u group than in the OVA group (Fig 6B). No significant difference in BALF macrophage numbers was detected among the mice from the OVA, HBoV-VP1u and B19V-VP1u groups (Fig 6D). Moreover, the activities of MMP-9 and MMP-2 in the BALF were analyzed. Significantly higher MMP-9 activities were detected in mice from the OVA, HBoV-VP1u and B19V-VP1u groups than in mice from the PBS group (Fig 7A). Notably, significantly higher MMP-9 activities were detected in mice from the HBoV-VP1u and B19V-VP1u groups than in mice from the OVA group (Fig 7A). Similar results were obtained with respect to MMP-2 activity. Significantly higher MMP-2 activities were observed in mice from the HBoV-VP1u and B19V-VP1u groups than in mice from the PBS and OVA groups (Fig 7A). The infiltration of lymphocytes into lung tissues was evaluated with H&E staining. Significantly greater lymphocyte infiltration was observed in mice from the OVA, HBoV-VP1u and B19V-VP1u groups than in mice from the PBS group (Fig 7B). Significantly greater lymphocyte infiltration was observed in mice from the HBoV-VP1u and B19V-VP1u groups than in mice from the OVA group (Fig 7B).

## Discussion

Human parvovirus B19 (B19V) and human bocavirus (HBoV) are important pathogenic parvoviruses that have been associated with a variety of human disorders [24]. Many global reports have indicated that there are associations between respiratory diseases and HBoV or B19V [10–14, 25–26]. However, the influence of B19V-VP1u and HBoV-VP1u on the development of asthmatic symptoms is still obscure. The current study is the first to reveal significant increases in the following parameters in OVA-sensitized mice from the HBoV-VP1u and B19-VP1u groups compared to those in mice from the OVA group: serum Penh ratios, IgE and Th2-biased cytokine (IL-4, IL-5, IL-10 and IL-13) levels; BALF OVA-specific IgE, IgG1 and IgG2a levels, MMP-9 and MMP-2 activities; and lymphocyte infiltration, along with significantly lower IgG2a levels. Thus, both the B19V-VP1u and HBoV-VP1u recombinant proteins increase the asthma-like symptoms in OVA-sensitized mice.

There is no animal model for human parvovirus B19V and HBoV, and these viruses can only be grown in culture with difficulty [27–28]. Hence, an alternative method that can mimic the symptoms induced by human parvovirus infection was sought. Accordingly, various studies have indicated that the subcutaneous injection of recombinant B19V-VP1u proteins into mice induces myocardial injury, dilated cardiomyopathy and lung injury, which are recognized as a model for the systemic effects of parvovirus infection [14, 29]. Notably, a study of a dilated cardiomyopathy mouse model that is generated with the subcutaneous injection of B19V-VP1u reported that siderophages, known as heart failure cells, were observed in lung tissues [29]. This is because macrophages engulf leaky red blood cells and accumulate in the lung tissue, which provides a possible explanation for the pathogenic mechanism of B19-VP1u in lung tissues. Accordingly, the subcutaneous injection of B19V-VP1u or HBoV-VP1u recombinant proteins is used to mimic the systemic effects of parvovirus infection to study the influence of the B19V-VP1u and HBoV-VP1u recombinant proteins on asthmatic symptoms in OVA-sensitized mice.

Most recent studies on phospholipase A (PLA) 2 have focused on its activity in inflammatory processes [30]. PLA2 is secreted from human macrophages and induces enzymes and cytokines that play important roles in inflammation and tissue damage, especially in the lung [31]. Various types of secretory PLA2 (sPLA2) are expressed in lung tissue and cause pulmonary edema [32]. Similarly, sPLA2 can induce acute lung injuries [33–34]. In patients with trauma, the serum level of sPLA2 is related to systemic inflammation, which compromises both the lung function and clinical status [35]. The VP1-unique (VP1u) regions of both B19V and HBoV have been shown to exhibit PLA2-like activity and are known to play essential roles in viral infectivity [36–37]. Moreover, the PLA2-like activity of B19V-VP1u and HBoV-VP1u has been implicated in a wide range of pathogenic responses, such as autoimmune diseases, cardiovascular disorders and respiratory diseases [7, 9–10, 38–40]. Our recent studies revealed a disruptive effect of both HBoV-VP1u and B19-VP1u on tight junctions in A549 cells [7] and demonstrated the induction of lung injury by both B19V-VP1u and HBoV-VP1u in naïve mice through the differential regulation of the NF-κB and MAPK signaling pathways [14]. These findings are similar to those of an earlier study in which epithelia in the respiratory tract were vulnerable to PLA2 as a result of its proteolytic activity [41]. Nevertheless, more direct evidence is required to verify the role of sPLA2 activity in relation to HBoV-VP1u and B19-VP1u in asthmatic pathogenesis.

Ovalbumin (OVA)-induced allergic asthma is currently the most commonly used model of induced asthma in a mouse. OVA induces an obvious T helper cell (Th) 2-biased immunological response [42–44]. Although OVA-induced asthma differs somewhat from human asthma, OVA-induced asthmatic mice exhibit various key features of clinical asthma, such as elevated IgE levels, airway inflammation, goblet cell hyperplasia, epithelial hypertrophy, AHR and bronchoconstriction [45]. Indeed, evidence shows that Th2 cytokines, such as IL-4, IL-5, IL-6, and IL-13, regulate B cell- and eosinophil-mediated responses, leading to IgE synthesis [46–48]. Notably, the presence of anti-parvovirus B19 IgE has been reported in patients with a B19V infection and has been associated with elevated mRNA levels of IL-4 and IL-10 [49]. Some data also suggest that HBoV- and B19V-specific IL-13 immune responses contribute to respiratory symptoms in patients with asthma [50]. In this study, significantly higher levels of IL-4, IL-5, IL-10 and IL13 were detected in both the serum and BALF of mice from the HBoV-VP1u and B19V-VP1u groups than in mice from the OVA group. These findings suggest a role for HBoV-VP1u and B19-VP1u in increasing Th2-biased cytokine levels and suggest a possible link between human parvoviruses and asthmatic symptoms.

Elevated allergen-specific IgE and IgG1 levels and a decreased IgG2 level are known hallmarks of asthma in humans [51–53]. A much lower level of IgG2 is detected in asthma patients than in healthy controls, whereas a higher IgG1 level is detected, perhaps due to Th2-biased cytokine expression in asthma, such as IL-4, which powerfully promotes immunoglobulin class-switching to IgG1 [53–55]. Similar results were observed in this study, including the significantly increased IgG1 and Th2 cytokine levels and decreased IgG2a levels that were detected in mice from the OVA, HBoV-VP1u and B19V-VP1u groups compared to those in the PBS group. Notably, the total IgG levels in the BALF of mice from the BoV-VP1u and B19V-VP1u groups were significantly lower than those in the BALF of mice from the OVA group. Since a lower total allergen-specific IgG content reflects a greater frequency of allergen exposure [56], the significantly decreased BALF total IgG in mice from the HBoV-VP1u and B19V-VP1u groups is likely to have been caused by a higher frequency of exposure to allergens. However, further investigations are required to verify this assumption.

Asthma is a life-long chronic disease that has been associated with a range of lung disorders. Resistive breathing in asthma causes large negative intrathoracic pressures and induces high-permeability pulmonary edema and lung inflammation [57]. A longitudinal, prospective study found that children with severe asthma have an increased risk of developing chronic obstructive pulmonary disease (COPD) [58]. Th2-biased cytokines and matrix metalloproteinases (MMPs) are the factors that are responsible for the progressive subepithelial fibrosis and structural changes in the extracellular matrix (ECM) in asthma [59]. Increased levels and activities of matrix metallopeptidase (MMP)-2 and MMP-9 have been observed in sputum or BALF from asthma patients compared to those from controls, and these increases were proportional to the severity of the airway inflammation and the extent of airway remodeling [59–60]. Notably, the present study reported increased asthmatic indices and elevated airway inflammation in OVA-sensitized mice treated with the HBoV-VP1u and B19V-VP1u recombinant proteins than in mice treated with OVA alone. These findings suggest a role for the human parvovirus VP1u protein in asthma patients who may have an increased risk for developing lung diseases.

## Conclusions

This study is the first to report the ability of human parvovirus VP1u to enhance asthmatic symptoms with a nonlocal viral infection method. This enhancement is suggested to be due to the sPLA2-like activity within the VP1u region and the increased induction of Th2-biased cytokines.

## Supporting information

S1 Fig The level of IFN-γ. The level of IFN-γ in (A) serum and (B) BALF of mice from PBS, OVA, HBoV-VP1u and B19V-VP1u groups is measured with ELISA kits. Values are mean ± SD. Similar results were observed in three repeated experiments. * and & indicate significant difference, p<0.05, relative to PBS and OVA groups, respectively.
(TIF)
