# Supplementary figures and images for: The effects of human parvovirus VP1 unique region in a mouse model of allergic asthma
Source: PLoS One. 2019 May 14;14(5):e0216799. doi: 10.1371/journal.pone.0216799 (PMC6516678; doi:10.1371/journal.pone.0216799)

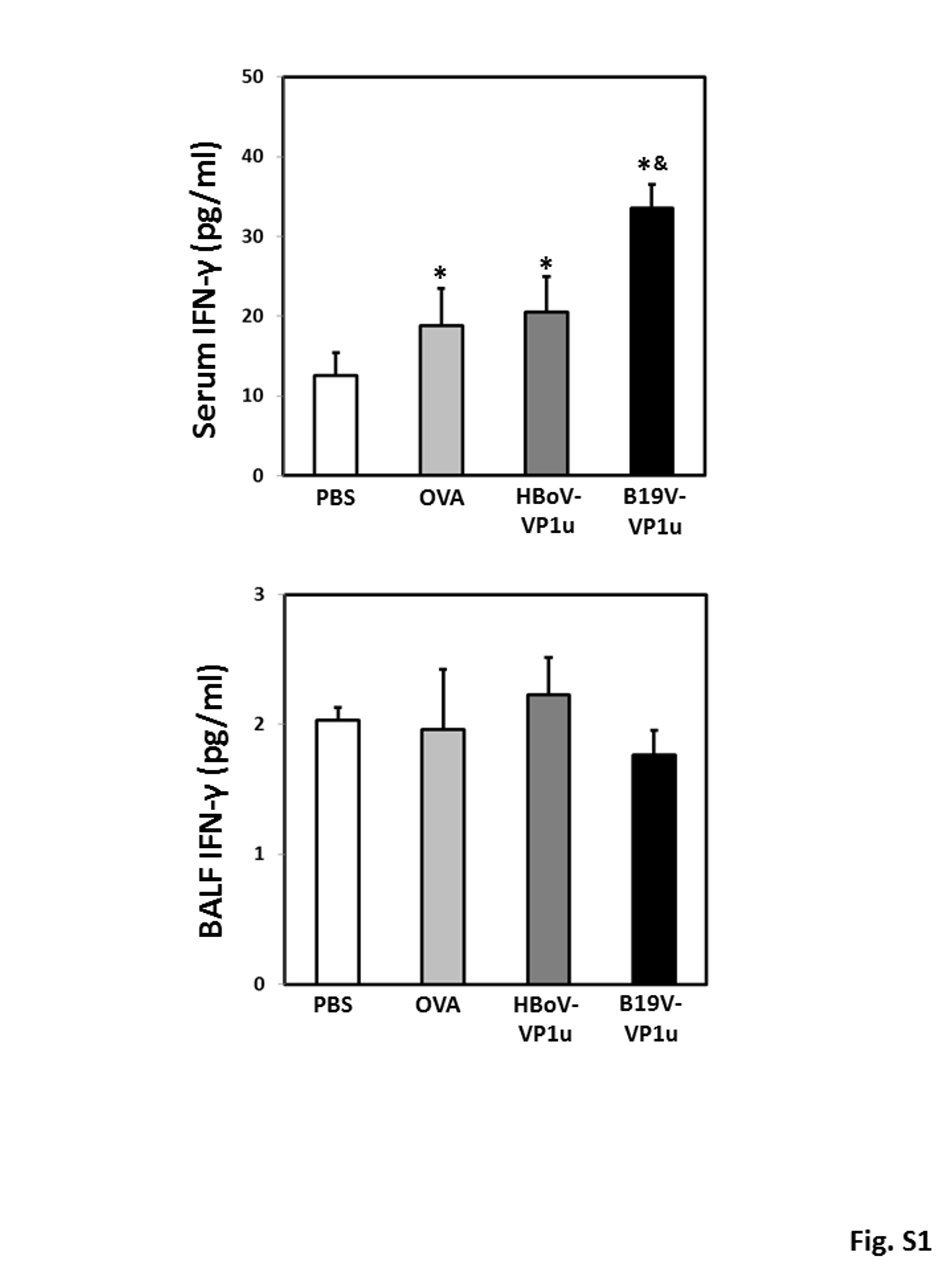

Supplement: S1 Fig — The level of IFN-γ in (A) serum and (B) BALF of mice from PBS, OVA, HBoV-VP1u and B19V-VP1u groups is measured with ELISA kits. Values are mean ± SD. Similar results were observed in three repeated experiments. * and & indicate significant difference, p<0.05, relative to PBS and OVA groups, respectively. (TIF) [file pone.0216799.s001.TIF]
